# Acute hypobaric hypoxia attenuates the anti-fatigue effects of pitolisant by downregulating the expression of organic cation transporter 1 and P-glycoprotein

**DOI:** 10.3389/fphar.2025.1564174

**Published:** 2025-04-16

**Authors:** Gang Zhao, Yajuan Li, Mengyuan Liu, Wei Chang, Hui Shen, Junhui Xue, Fengzhou Liu

**Affiliations:** ^1^ Aerospace Clinical Medicine Center, Air Force Medical University, Xi’An, China; ^2^ Northwestern University School of Medicine, Xi’An, China; ^3^ Department of Neurosurgery, Second Affiliated Hospital of Air Force Medical University, Xi’An, China; ^4^ Aviation Medicine Department, First Affiliated Hospital of Air Force Medical University, Xi’An, China; ^5^ Innovation Research Institute, Xijing Hospital, Air Force Medical University, Xi’an, China

**Keywords:** pitolisant, hypobaric hypoxia, pharmacodynamics, drug transporters, LC-MS/MS

## Abstract

**Objective:**

This study investigates the effects of acute hypobaric hypoxia (HH) on the anti-fatigue properties of pitolisant and explores the underlying mechanisms. The aim is to provide a theoretical basis for expanding its clinical indications and optimizing its use in individuals exposed to HH conditions.

**Methods:**

The anti-fatigue effects of pitolisant were evaluated using the water maze, novel object recognition, and rotating rod tests. Drug concentrations and dopamine levels were analyzed using High-Performance Liquid Chromatography-Mass Spectrometry/Mass Spectrometry (HPLC-MS/MS). Additionally, gene and protein expression levels of P-glycoprotein (P-gp) and organic cation transporter 1 (OCT1) were assessed to explore the mechanisms by which HH affects pitolisant’s pharmacodynamics.

**Results:**

A 40 mg/kg dose of pitolisant significantly improved learning, memory, cognitive, and motor functions in sleep-deprived mice under HH conditions (*P* < 0.05). Pharmacokinetic analysis revealed a reduction in pitolisant concentration in the brain under HH conditions. Furthermore, OCT1 protein expression decreased after 1 h and 1 day of HH exposure (*P* < 0.05), while P-gp expression decreased after 1 h (*P* < 0.05).

**Conclusion:**

HH possibly reduced pitolisant’s brain concentration and efficacy by altering the expression of OCT1 and P-gp transporters. A 40 mg/kg dose was necessary for an effective anti-fatigue response. Pitolisant shows potential for supporting circadian rhythm regulation in shift workers and individuals suffering from jet lag. When used under HH conditions, adjusting the dose and frequency may be necessary due to altered pharmacokinetics.

## 1 Introduction

Pitolisant, a novel histamine H_3_ receptor antagonist, promotes wakefulness and enhances cognitive function by blocking H_3_ receptors, thereby increasing the release of endogenous histamine in the central nervous system. It has been shown to be highly effective in treating excessive daytime sleepiness and cataplexy, with its clinical indications primarily being narcolepsy (Dauvilliers et al., 2013; Meskill et al., 2022). Studies have also suggested that pitolisant may alleviate daytime sleepiness in patients with obstructive sleep apnea (Dauvilliers et al., 2020). However, there is limited information regarding its anti-fatigue effects in conditions associated with circadian rhythm disruption, such as overtime, shift work, or continuous work.

The high-altitude environment, characterized by low atmospheric pressure, hypoxia, intense radiation, and cold, dry conditions, significantly impacts human physiological functions, with hypobaric hypoxia (HH) being the primary factor. A HH environment not only induces physiological adaptive responses to cope with environmental stress but may also alter the biological processes of drugs within the body, thereby affecting their efficacy and safety. The effects and mechanisms of central nervous system (CNS) drugs in HH environment, however, remain insufficiently explored. Therefore, investigating the changes in drug pharmacodynamics and the underlying mechanisms in HH is crucial for ensuring the rational use of medications in high-altitude areas.

Previous studies have shown that HH environments can lead to alterations in the structure and function of the blood-brain barrier (BBB), which may hinder drug transport across the BBB and subsequently affect their therapeutic effects in the CNS (Langen et al., 2019; Bonkowsky and Son, 2018). Drug transporters play a key role in determining the absorption, distribution, and barrier-crossing transport of drugs (Jala et al., 2021). Zolotoff et al. (2020) reported using an *in vitro* BBB model that chronic intermittent hypoxia for 6 h could impair the structure and function of the BBB, thereby reducing the efficiency of drug transport. Li et al. (Duan et al., 2021) revealed that changes in drug transporter function in HH environments could alter the absorption and metabolism of drugs such as vincristine, consequently altering their therapeutic effects. Therefore, understanding how HH influences drug pharmacodynamics and its potential mechanisms is vital for guiding rational drug use in high-altitude areas and ensuring the health of travelers, business personnel, and military forces in rapidly ascending regions.

Therefore, this study aims to clarify the anti-fatigue effects of different doses of pitolisant in 24-h sleep-deprived mice, and to explore the pharmacodynamic changes and potential mechanisms of pitolisant in HH environment. This research intends to expand the clinical indications of pitolisant and provide theoretical support and practical guidance for its rational use in HH environments.

## 2 Materials and methods

### 2.1 Animals

Male C57BL/6J mice (SPF grade), aged 6–8 weeks and weighing 20–25g (purchased from the Animal Experiment Center, Air Force Military Medical University), were housed in the same institution under standard laboratory conditions. Mice were provided with *ad libitum* access to food and water, maintained on a 12-h light/dark cycle, and kept at a temperature of approximately 25°C. All animal experiments were approved by the Animal Ethics Committee of the Air Force Military Medical University.

### 2.2 Instruments

The LC-MS/MS system consisted of an high-performance liquid chromatography (HPLC) system (Waters, United States) coupled with an ACQUITY QDa Detector single quadrupole mass spectrometer (Waters, United States). Additional equipment included a small animal hypobaric chamber (custom-built by the department), a tissue homogenizer (Seville, China), a modified multi-water platform environmental sleep deprivation chamber (customized), a −80°C ultra-low temperature freezer (Haier, China), a Western blot chemiluminescence detection system (Vilber Fusion FX Spectra, France), and a real-time quantitative PCR instrument (Bio-Rad, United States).

### 2.3 Reagents and drugs

Pitolisant (CAS: 362665-56-3, Aladdin) (The drug concentration in the text refers to the free form of pitolisant. In all experiments, pitolisant was administered as a single dose.), chromatographic acetonitrile (CAS: 75-05-8, Fisher), chromatographic methanol (CAS: 67-56-1, Fisher), dopamine (CAS: 62-31-7, Shanghai YuanYe Biotechnology Co., Ltd.), GAPDH antibody (CAS: 60004-1-1g, ProteinTech), P-gp antibody (CAS: AB170904, Abcam), OCT1 antibody (CAS: AF0224, Affinity), ECL chemiluminescent reagent (CAS: IC-8001-100, InCellGen), Western blot pre-cast gels (CAS: IC-8208, InCellGen), reverse transcription kit (CAS: DY10502, Diagenode), and PCR reagent kit (CAS: DY20301, Diagenode).

### 2.4 Sleep deprivation model

The modified multi-platform system consisted of a water tank and platforms elevated 1 cm above the water surface. Mice were able to stand on 2.5 cm diameter platforms. Upon entering a sleep state, the reduction in muscle tone caused rhythmic head drops and contact with the water, thereby inducing the sleep deprivation model.

### 2.5 HH model

Mice were placed in a hypobaric chamber and exposed to a rapid ascent (10–15 m/s) to an altitude of 5,000 m, where the chamber pressure was maintained at 405 mmHg ±6 mmHg for 24 h to simulate HH environment.

### 2.6 Morris water maze task and grouping

The water maze used in this experiment had a diameter of 1.2 m. Behavior was recorded and analyzed using the SMART V3.0 software and corresponding camera system. The spatial navigation task was conducted over four consecutive days. The platform was positioned in the first quadrant, and each day, mice underwent four training trials with at least 1 h intervals between trials. The first trial began in the first quadrant, followed by the second, third, and fourth quadrants on the first day. On subsequent days, training began in the next quadrant in sequence. The time taken by the mice to find the platform within 60 s was recorded as the escape latency. If a mouse successfully located the platform, it was allowed to stay on the platform for 5 s before being removed, dried, and returned to the cage. If the mouse failed to find the platform within 60 s, the escape latency was recorded as 60 s, and the mouse was gently guided to the platform, where it stayed for 15 s before the same procedure was repeated. During the 4 days of training, HH group mice were immediately returned to the hypobaric chamber after each training session. On the fifth day, prior to the formal spatial exploration test, mice were gavaged 0.5 h before the experiment. The platform located in the first quadrant was removed, and the mice were placed in the third quadrant on the opposite side. The time taken to find the original platform, the number of times the mice crossed the location of the original platform, and the time spent in the original platform quadrant were recorded and analyzed.

Experimental Grouping: 6-8-week-old male C57BL/6J mice were randomly assigned to ten groups: Control group, Placebo + SD24 h group, 5 mg/kg pitolisant + SD24 h group, 20 mg/kg pitolisant + SD24 h group, 40 mg/kg pitolisant + SD24 h group, HH group, HH + SD24 h group, 5 mg/kg pitolisant + HH + SD24 h group, 20 mg/kg pitolisant + HH + SD24 h group, and 40 mg/kg pitolisant + HH + SD24 h group, with 5 mice per group.

### 2.7 Novel object recognition task and grouping

The novel object recognition task capitalizes on the innate tendency of rodents to explore unfamiliar objects, and it is used to assess cognitive function by recording the amount of time spent exploring novel and familiar objects. Mice were placed in a 40 cm × 40 cm × 40 cm container, where two identical cone-shaped objects were positioned. The mice were allowed to explore the objects freely for 5 min. After at least 1 h, one of the cone-shaped objects was replaced with a new object, and the mouse was returned to the container for another 5 min of free exploration. Throughout the experiment, the time spent sniffing the objects or interacting within 2 cm of the object surface was continuously monitored and recorded. The cognitive index (CI) was calculated using the formula:

$$\text{CI}=\left(\text{Time spent exploring the novel object}\right)\;/\;\left(\text{Time spent exploring the novel}+\text{familiar object}\right)\times 100\%.$$

Experimental Grouping:6-8-week-old male C57BL/6J mice were randomly assigned to one of eight groups: Control group, Placebo +SD24 h group, 20 mg/kg pitolisant +SD24 h group, 40 mg/kg pitolisant +SD24 h group, HH group, HH + SD24 h group, 20 mg/kg pitolisant + HH + SD24 h group, and 40 mg/kg pitolisant + HH + SD24 h group, with 5 mice per group.

### 2.8 Rotarod test

The rotarod test is a widely used method to assess motor function in rodents. It is also employed in fatigue experiments and for evaluating the effects of drugs on motor performance and their potential side effects. During the experiment, if an animal falls from the rod, the system automatically records the duration of time the animal remained on the rod, as well as the speed of the rod at the moment of the fall. On Day 1, the animals underwent an adaptation session at a speed of 5 rpm for 5 min per trial, with three trials in total. Mice with clear motor coordination deficits were excluded from the study. On Day 2, training began at 5 rpm, with the speed increasing to 10 rpm over a 300-s period. This was repeated for three trials, each lasting 5 min, with at least 30 min of rest between trials for fatigue recovery. On Day 3, the procedure was similar, but the speed was increased to 20 rpm over 300 s, and again, three trials were conducted. On Day 4, the formal test began at 5 rpm, with the speed increasing to 40 rpm within 90 s. The time to fall and the speed of the rod at the moment of the fall were recorded.

Experimental Grouping: The experimental groups were the same as those used in the novel object recognition test.

### 2.9 Dose selection

The dose selection was based on previous studies (Kitanaka et al., 2020; Kotańska et al., 2020; Krief et al., 2021; Uguen et al., 2013; Abdurakhmanova et al., 2020), and the choice of three gradient concentrations was aimed at identifying the optimal dose (i.e., a dose that maximizes efficacy without significant toxic side effects) for the subsequent pharmacokinetic study.

### 2.10 LC-MS conditions

For the measurement of pitolisant plasma and brain concentrations, a dose of 40 mg/kg of pitolisant was used.

#### 2.10.1 LC-MS conditions for blood drug concentration measurement

Chromatographic separation was achieved using a CORTECS C18 column (4.6 mm × 50 mm, 2.7 µm) at 30°C. The mobile phase consisted of ultrapure water and acetonitrile (30:70, v/v) and was eluted isocratically for 6 min at a flow rate of 0.8 mL/min. The injection volume was 3 μL, and detection was conducted at 220 nm. Mass spectrometric detection was performed with an electrospray ionization (ESI) source in positive ion multiple reaction monitoring (MRM) mode. The precursor ion for pitolisant was 296.3 m/z. The cone voltage was set at 20 V, capillary voltage at 0.8 kV, and probe temperature at 600°C.

#### 2.10.2 LC-MS conditions for brain tissue drug concentration measurement

Chromatographic separation was achieved using a CORTECS C18 column (4.6 mm × 50 mm, 2.7 µm) at 30°C. The mobile phase consisted of ultrapure water and acetonitrile (70:30, v/v) and was eluted isocratically for 3 min at a flow rate of 0.8 mL/min. The injection volume was 3 μL, and detection was conducted at 220 nm. Mass spectrometric detection utilized an electrospray ionization (ESI) source in positive ion multiple reaction monitoring (MRM) mode, with the precursor ion for pitolisant at 296.3 m/z. The cone voltage was 20 V, capillary voltage at 0.8 kV, and probe temperature at 600°C.

#### 2.10.3 LC-MS conditions for dopamine measurement in brain tissue

Chromatographic separation was performed using a CORTECS C18 column (4.6 mm × 50 mm, 2.7 µm) at 30°C. The mobile phase consisted of 0.1% formic acid in water and acetonitrile (70:30, v/v) and was eluted isocratically for 2 min at a flow rate of 0.5 mL/min. The injection volume was 3 μL, and detection was carried out at 254 nm. The mass spectrometer was equipped with an electrospray ionization (ESI) source in positive ion multiple reaction monitoring (MRM) mode, with dopamine detected at 154 m/z. The cone voltage was set at 20 V, capillary voltage at 0.8 kV, and probe temperature at 600°C.

#### 2.10.4 Plasma sample collection and processing

Blood samples were collected from the retro-orbital venous plexus of the mice and transferred into 1.5 mL centrifuge tubes containing an anticoagulant. After standing at room temperature for 0.5 h, the samples were centrifuged at 8,000 rpm and 4°C for 15 min. Plasma (100 µL) was collected and mixed with 200 µL acetonitrile. The mixture was vortexed and centrifuged at 8,000 rpm at 4°C for 10 min. The supernatant was then collected for analysis.

#### 2.10.5 Brain tissue sample collection and processing

Mice were euthanized by cervical dislocation, and their brains were rapidly removed on ice. The brain tissue was precisely weighed and homogenized in 2 volumes of 0.2% formic acid in water (pre-chilled on ice). After homogenization, the mixture was centrifuged at 8,000 rpm at 4°C for 15 min. The supernatant (100 µL) was mixed with 200 µL acetonitrile, vortexed, and centrifuged at 8,000 rpm at 4°C for 15 min. The supernatant was then collected for analysis.

#### 2.10.6 Preparation of standard solutions

##### 2.10.6.1 Preparation of plasma standard solutions for drug concentration measurement

The pitolisant stock solution was prepared at a concentration of 10 μg/mL. This stock solution was serially diluted with blank mouse plasma to prepare plasma samples with final concentrations of 5, 10, 50, 100, and 1,000 ng/mL.

##### 2.10.6.2 Preparation of brain tissue standard solutions for drug concentration measurement

The pitolisant stock solution was prepared at a concentration of 10 μg/mL. This stock solution was serially diluted with blank brain tissue supernatant to prepare standard solutions with final concentrations of 5, 10, 50, 100, 500, and 1,000 ng/mL.

##### 2.10.6.3 Preparation of dopamine standard solutions

A precise amount of 5 mg of dopamine was dissolved in a 0.1% formic acid solution to prepare a dopamine stock solution with a concentration of 5 mg/mL. The dopamine stock solution was then serially diluted to obtain standard solutions with final concentrations of 5, 50, 100, 500, 1,000, and 5,000 ng/mL.

### 2.11 Statistical analysis

Statistical analyses were performed using Student’s t-test and one-way analysis of variance (ANOVA). A p-value of <0.05 was considered statistically significant. All statistical calculations were conducted using GraphPad Prism software.

## 3 Results

### 3.1 Effects of pitolisant on learning, memory, cognitive function, and motor coordination in mice

#### 3.1.1 Pitolisant significantly improved learning and memory abilities in mice subjected to sleep deprivation under HH conditions

**FIGURE 1:**
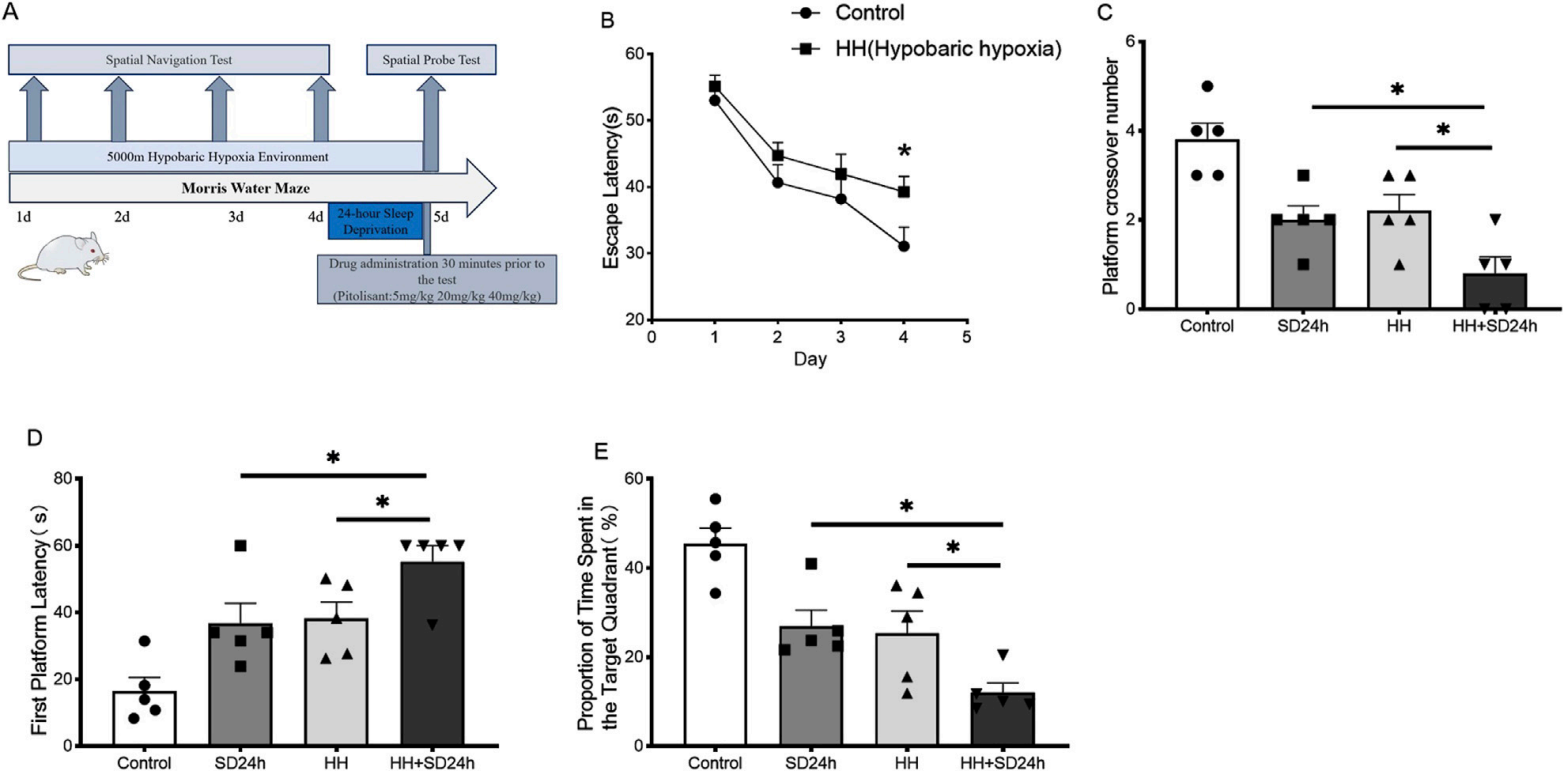
Effects of HH on learning and memory performance in mice following 24 h of sleep deprivation. **(A)** Flowchart of the morris water maze protocol; **(B)** Escape latency during the first 4 days of the spatial navigation training; **(C)** Number of crossings over the original platform in the spatial probe test; **(D)** Time to first platform location retrieval in the spatial probe test; **(E)** Percentage of time spent in the target quadrant during the spatial probe test; *P < 0.05, n = 5. SD24 h, 24-h sleep deprivation; HH, Hypobaric hypoxia; HH + SD24 h, Hypobaric hypoxia + 24-h sleep deprivation.

During the training phase of the Morris water maze task (Figure 1A), the escape latency progressively shortened as the number of training trials increased in all groups. Compared to the control group, the escape latency of the HH group was significantly prolonged on the fourth day of training (Figure 1B) (*P* < 0.05). In the spatial exploration test, compared to the control group, both the SD24 h and HH groups of mice exhibited a significant decrease in the number of platform crossings (Figure 1C) (*P* < 0.05), required more time to locate the platform for the first time (Figure 1D) (*P* < 0.05), and spent less time in the target quadrant (Figure 1E) (*P* < 0.05). The HH + SD24 h group displayed more pronounced memory impairments (Figures 1C–E) (*P* < 0.05). These results suggest that exposure to HH conditions exacerbates the learning and memory deficits caused by 24-h sleep deprivation in mice.

**FIGURE 2:**
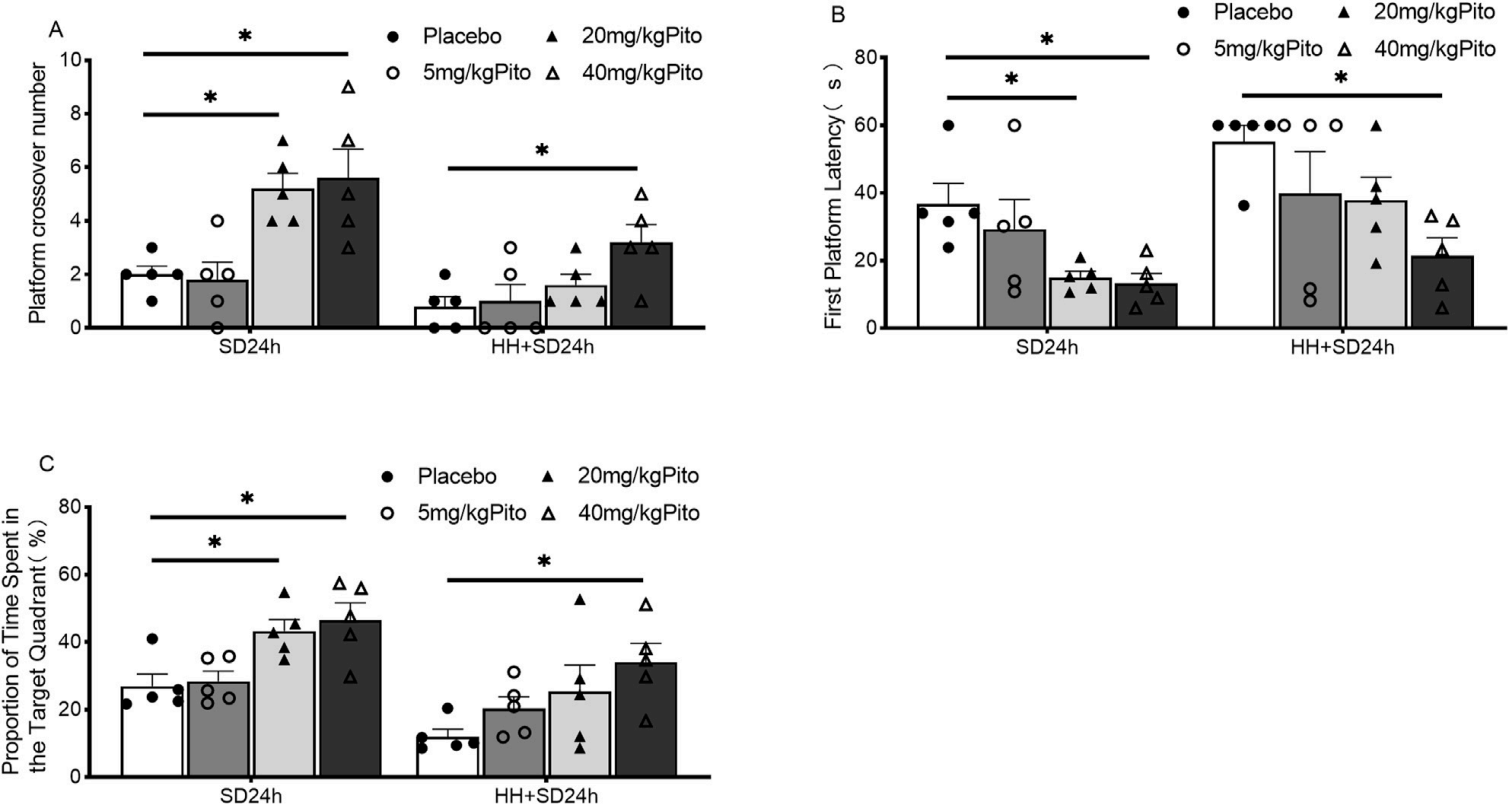
Effects of different doses of pitolisant on learning and memory in mice subjected to 24 h of sleep deprivation under HH conditions. **(A)** Number of crossings over the original platform in the spatial probe test; **(B)** Time to first platform location retrieval in the spatial probe test; **(C)** Percentage of time spent in the target quadrant during the spatial probe test; *P < 0.05, n = 5. SD24 h, 24-h sleep deprivation; HH + SD24 h, Hypobaric hypoxia +24-h sleep deprivation.

In the spatial exploration experiment, under normoxic conditions, compared to the SD24 h + placebo group, the 20 mg/kg and 40 mg/kg groups of mice showed a significant increase in the number of platform crossings (Figure 2A) (*P* < 0.05), required less time to find the platform for the first time (Figure 2B) (*P* < 0.05), and spent more time in the target quadrant (Figure 2C) (*P* < 0.05). No significant differences were observed between the 20 mg/kg and 40 mg/kg groups. Under HH conditions, compared to the HH + SD24 h + placebo group, no significant differences were observed in the number of platform crossings, time taken to find the platform for the first time, or time spent in the target quadrant for the 5 mg/kg and 20 mg/kg groups. However, the 40 mg/kg group showed a significant increase in the number of platform crossings (Figure 2A) (*P* < 0.05), required less time to locate the platform for the first time (Figure 2B) (*P* < 0.05), and spent more time in the target quadrant (Figure 2C) (*P* < 0.05). These results suggest that, under HH conditions, 40 mg/kg pitolisant significantly improves learning and memory in mice subjected to 24-h sleep deprivation.

#### 3.1.2 Pitolisant significantly improves cognitive function in mice subjected to sleep deprivation under HH conditions

**FIGURE 3:**
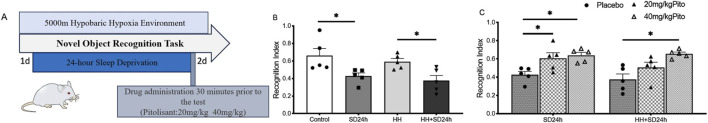
Effects of different concentrations of pitolisant on cognitive function in mice subjected to 24 h of sleep deprivation under HH conditions. **(A)** Flowchart of the novel object recognition task protocol; **(B)** Cognitive index of mice in the untreated group; **(C)** Cognitive index of mice in the treated groups. *P < 0.05, n = 5. SD24 h, 24-h sleep deprivation; HH, Hypobaric hypoxia; HH + SD24 h, Hypobaric hypoxia + sleep deprivation.

The procedure for the novel object recognition test is illustrated in (Figure 3B). The results showed that the cognitive index of mice in the SD24 h group was significantly lower than that of the control group (Figure 3B) (*P* < 0.05). Additionally, the cognitive index of mice in the HH + SD24 h group was significantly lower than that of the HH group (Figure 3B) (*P* < 0.05). Under normoxic conditions, mice in the 20 mg/kg and 40 mg/kg groups exhibited significantly higher cognitive indices compared to the SD24 h + placebo group (Figure 3C) (*P* < 0.05). However, under HH conditions, no significant difference was observed between the 20 mg/kg group and the HH + SD24 h + placebo group. In contrast, mice in the 40 mg/kg group displayed a significantly higher cognitive index than those in the HH + SD24 h + placebo group (Figure 3C) (*P* < 0.05). These findings suggest that 40 mg/kg pitolisant significantly improves cognitive function in mice subjected to SD24 h under HH conditions.

#### 3.1.3 Pitolisant significantly improves motor coordination in mice subjected to sleep deprivation under HH conditions

**FIGURE 4:**
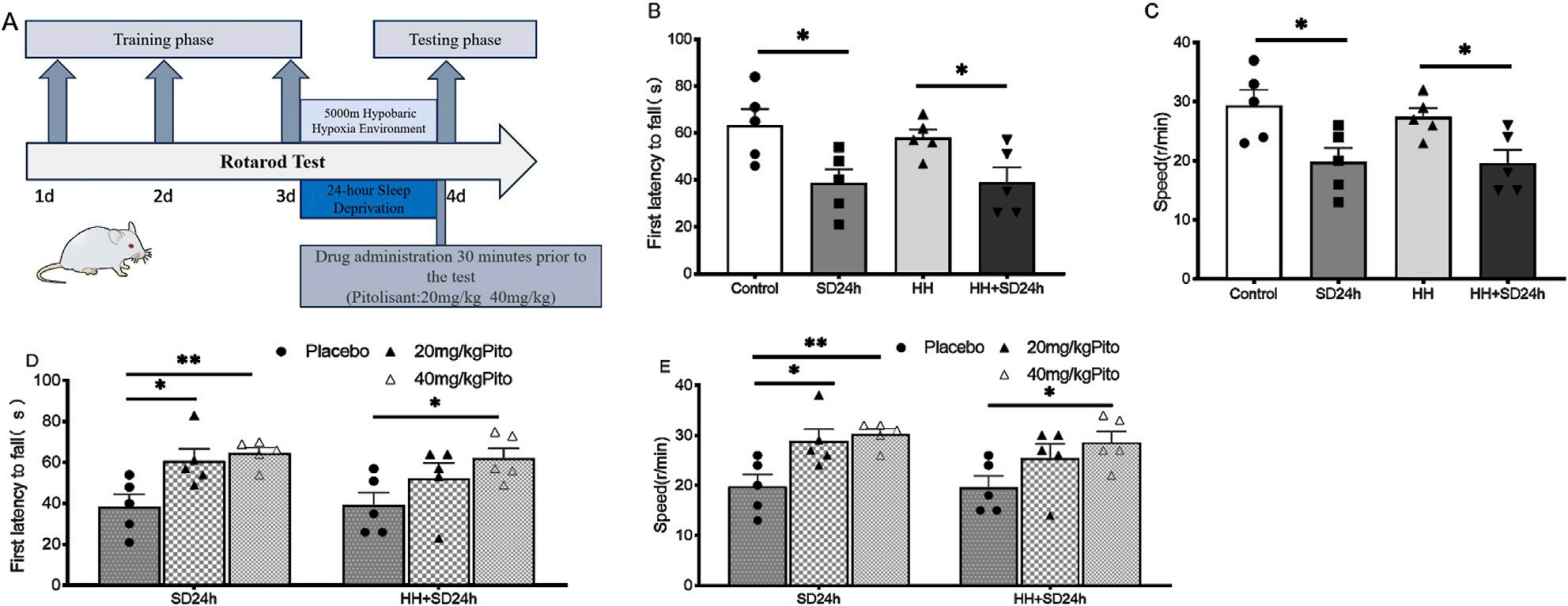
Effects of different concentrations of pitolisant on motor coordination in sleep-deprived mice under HH. **(A)** Flowchart of the rotarod test protocol; **(B)** Time to first fall from the rotarod in the group without pitolisant; **(C)** Rod rotation speed at the time of falling in the group without pitolisant; **(D)** Time to first fall from the rotarod in the group with pitolisant; **(E)** Rod rotation speed at the time of falling in the group with pitolisant. *P < 0.05, **P < 0.01, n = 5. SD24 h, 24-h sleep deprivation; HH, Hypobaric hypoxia; HH + SD24 h, Hypobaric hypoxia + sleep deprivation.

The procedure for the rotarod test is illustrated in (Figure 4A). The results showed that the time to first fall from the rod and the rod rotation speed at the time of falling were significantly lower in the SD24 h group compared to the Control group (Figure 4B) (*P* < 0.05). Similarly, mice in the HH + SD24 h group had significantly lower time to first fall and rod rotation speed than those in the HH group (Figure 4C) (*P* < 0.05). Under normoxic conditions, the time to first fall and rod rotation speed in the 20 mg/kg and 40 mg/kg groups were significantly higher than those in the SD24 h + Placebo group (Figures 4D, E) (*P* < 0.05). In contrast, under HH conditions, no significant differences were observed between the 20 mg/kg group and the HH + SD24 h + Placebo group; however, mice in the 40 mg/kg group showed significant improvements in both the time to first fall and rod rotation speed (Figures 4D, E) (*P* < 0.05). These results suggest that 40 mg/kg pitolisant significantly improves motor coordination in sleep-deprived mice under HH conditions.

### 3.2 No significant effect of pitolisant on dopamine levels in the brain of mice under HH conditions

**FIGURE 5:**
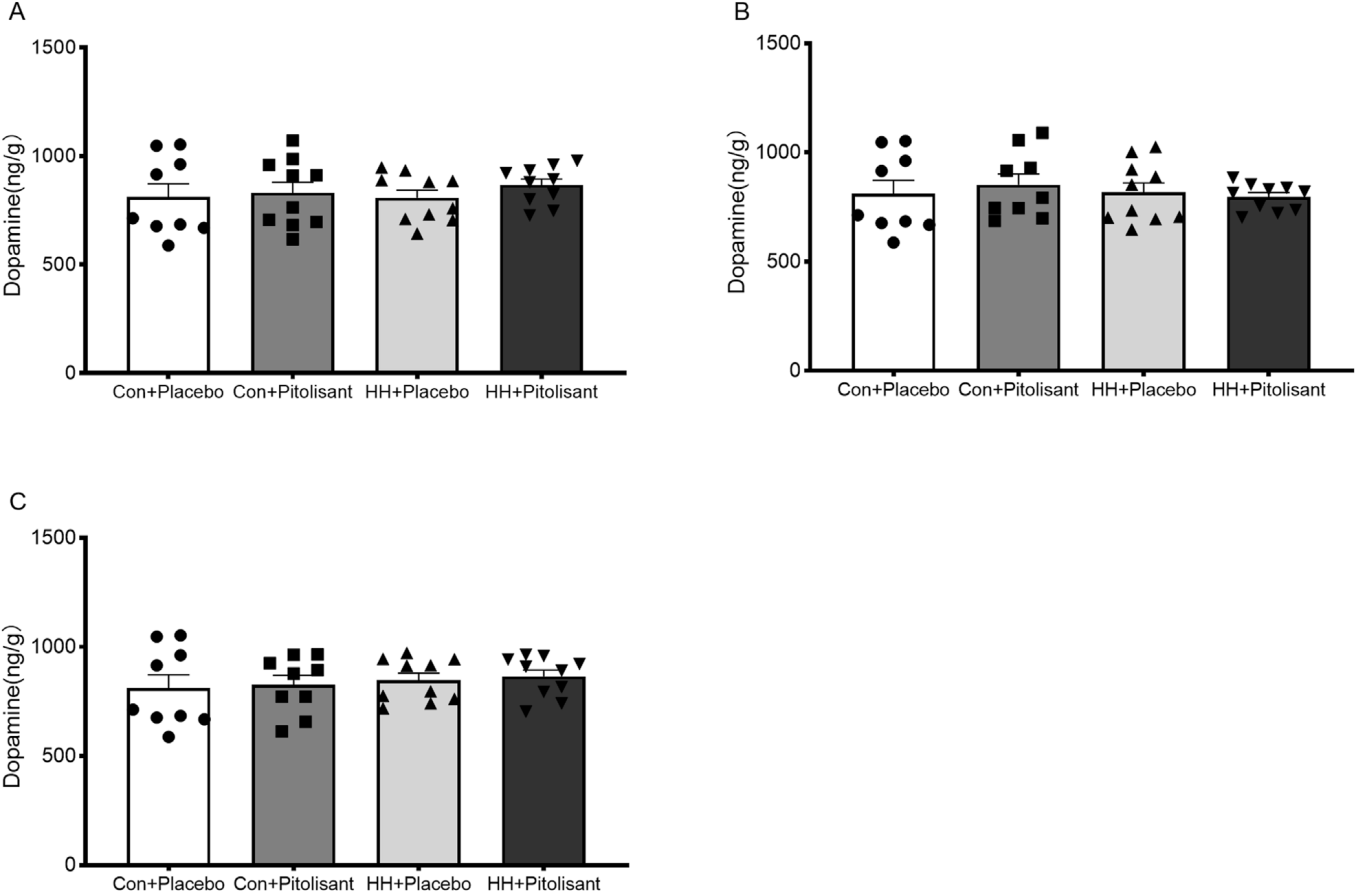
Effects of pitolisant on dopamine levels in the brain of mice. **(A)** Dopamine levels 1 h after pitolisant/placebo administration; **(B)** Dopamine levels 1 day after pitolisant/placebo administration; **(C)** Dopamine levels 3 days after pitolisant/placebo administration; n = 9–10.

To determine the effect of pitolisant on dopamine levels in the brain under HH conditions, dopamine concentrations were measured using HPLC-MS/MS. The results (Figures 5A–C) showed no significant difference in dopamine levels between the pitolisant and placebo groups at 1 h, 1 day, or 3 days after administration under HH conditions.

### 3.3 The drug concentration of pitolisant in the brain and plasma, and its brain penetration

**FIGURE 6:**
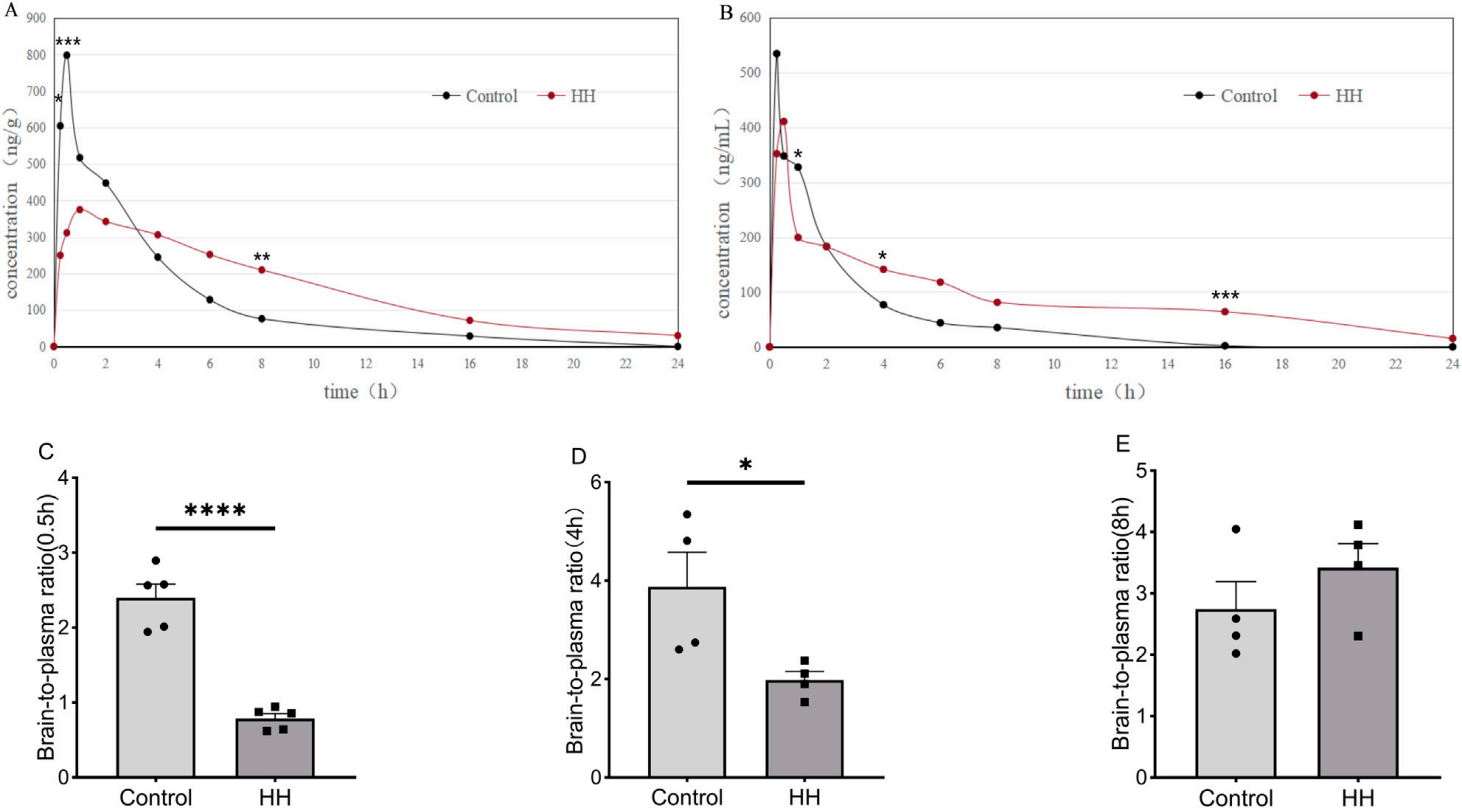
Pitolisant concentrations in blood and brain over time. **(A)** Brain drug concentration-time curve; **(B)** Blood drug concentration-time curve; **(C)** Brain-to-plasma ratio of pitolisant at 0.5 h; **(D)** Brain-to-plasma ratio of pitolisant at 4 h; **(E)** Brain-to-plasma ratio of pitolisant at 8 h. *P < 0.05, ***P < 0.001 vs. control group; n = 4–5.

Compared to the control group (Figure 6A), the HH group exhibited significantly lower brain drug concentrations during the absorption and distribution phase (at 0.25 and 0.5 h after administration) (*P* < 0.05). However, during the elimination phase (at 8 h post-administration), brain drug concentrations were significantly higher (*P* < 0.05). In the plasma (Figure 6B), the HH group had significantly lower drug concentrations at 1 h after administration (*P* < 0.05), but during the elimination phase, drug concentrations were significantly higher at 4 and 16 h administration (*P* < 0.05). We calculated the brain-to-plasma ratio (C_brain_/C_plasma_) of pitolisant at different time points after administration. The results indicated that under normoxic conditions, pitolisant exhibited the highest brain penetration of 3.875 at 4 h post-administration, whereas under HH conditions, the highest brain penetration of 3.418 was observed at 8 h post-administration. We conducted statistical analyses of pitolisant brain penetration during the peak concentration period (0.5 h)and at 4 h and 8 h post-administration in both the HH and normoxic groups. It was found that at 0.5 h and 4 h (Figures 6C, D), the brain penetration of pitolisant in the HH group was significantly lower than that in the control group (P<0.05), while no significant difference was observed between the two groups at 8 h (Figure 6E). These results suggest that HH significantly alters the absorption, distribution, and metabolism of pitolisant, reducing absorption and prolonging its metabolic elimination.

### 3.4 Effects of HH on the expression of P-gp and OCT1 genes and proteins in the brains of pitolisant-treated mice

**FIGURE 7:**
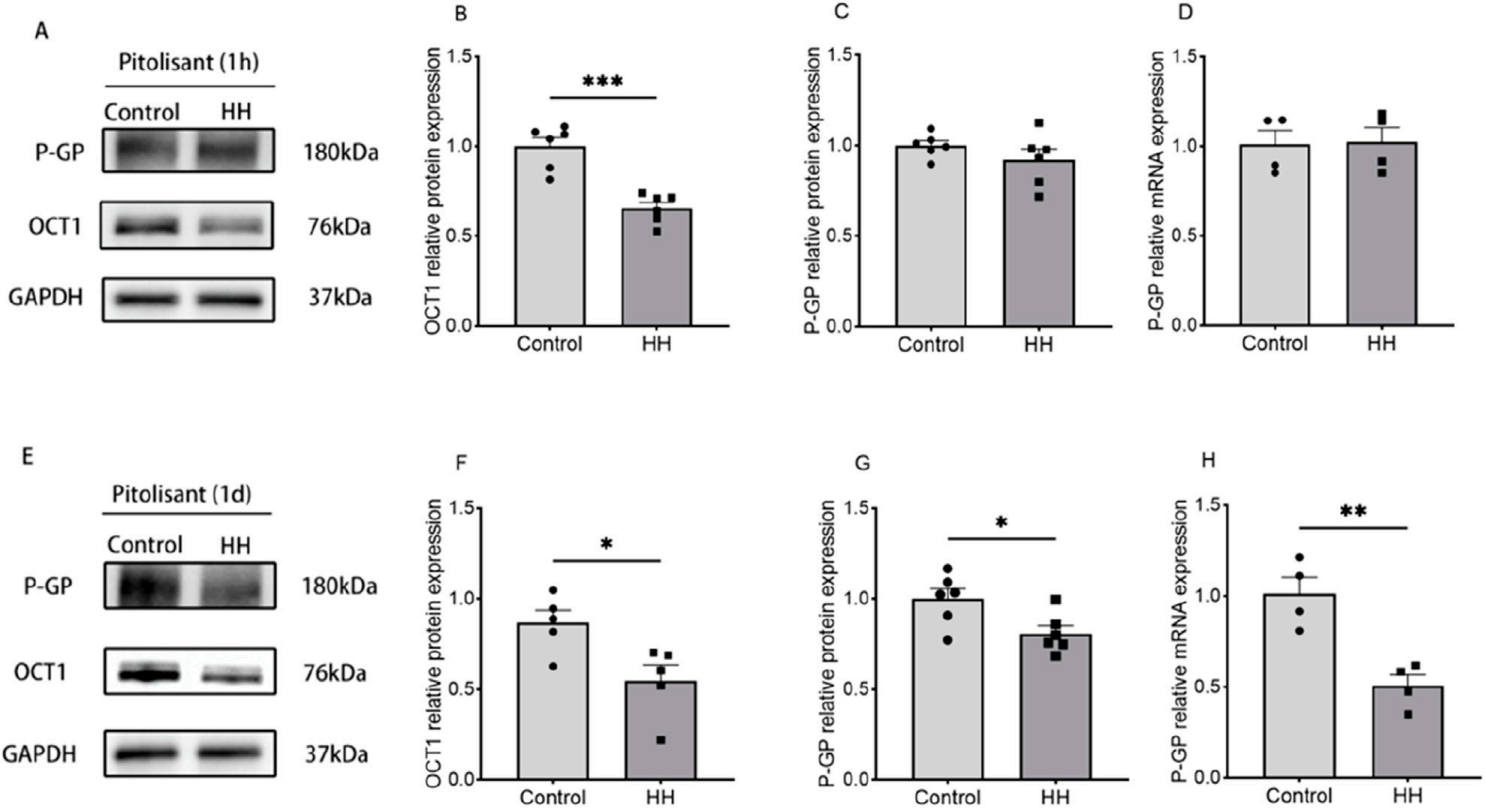
Expression of OCT1 protein and P-gp gene and protein. **(A)** Western blot results 1 h after pitolisant administration; **(B)** Relative expression of OCT1 protein 1 h after administration; **(C)** Relative expression of P-gp protein 1 h after administration; **(D)** Relative expression of P-gp mRNA 1 h after administration; **(D)** Relative expression of P-gp mRNA 1 h after administration. **(E)** Western blot results 1 day after administration. **(F)** Relative expression of OCT1 protein 1 day after administration. **(G)** Relative expression of P-gp protein 1 day after administration. **(H)** Relative expression of P-gp mRNA 1 day after administration.*P < 0.05, **P < 0.001, **P < 0.01, n = 4–6.

To investigate the mechanisms behind the reduced drug concentrations in the brain, the expression of P-gp and OCT1 transporters in the brain was analyzed using Western blot and qPCR after acute HH exposure for 1 h and 1 day. The results showed that after 1 h of HH, OCT1 protein expression was significantly decreased (Figure 7A, B) (*P* < 0.001), while P-gp gene and protein levels showed no significant changes (Figures 7C, D). After 1 day of HH exposure, both OCT1 protein (Figure 7E, F) (*P* < 0.05) and P-gp protein and gene expression (Figures 7G, H) (*P* < 0.05) were significantly reduced.

### 3.5 Methodological considerations

#### 3.5.1 Method specificity

**FIGURE 8:**
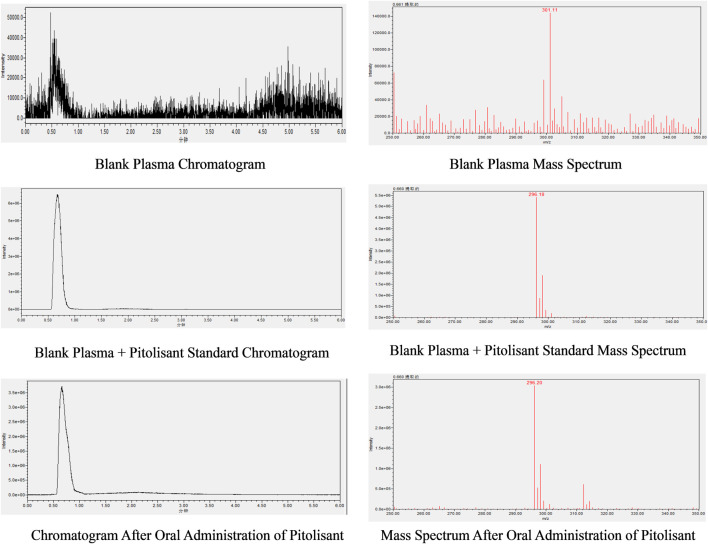
Chromatogram and mass spectrum of pitolisant in mouse plasma.

The chromatogram and mass spectrum of pitolisant in plasma (Figure 8) show a retention time of approximately 0.66 min, with no interfering peaks, well-formed peaks, and minimal baseline noise.

**FIGURE 9:**
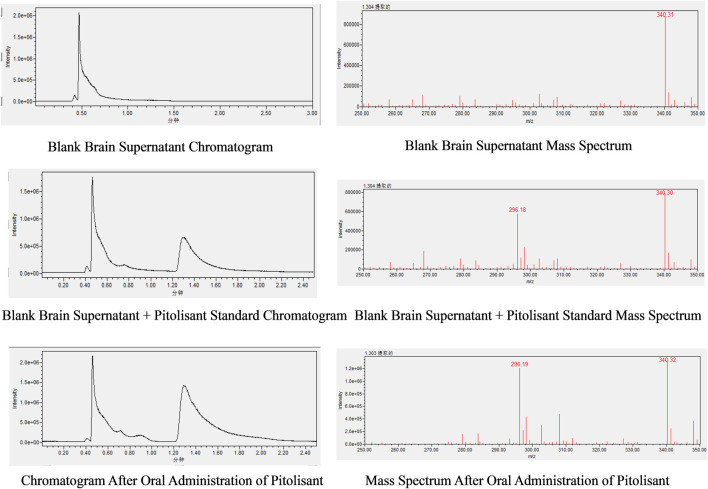
Chromatogram and mass spectrum of pitolisant in mouse brain homogenate.

The chromatogram and mass spectrum of pitolisant in brain homogenate (Figure 9) reveal a retention time of approximately 1.30 min, with complete separation from other components, well-defined peaks, and low baseline noise.

#### 3.5.2 Regression equations and detection limits

Regression equation and detection limit of pitolisant in plasma: A good linear relationship was observed for pitolisant concentrations ranging from 5 ng/mL to 1,000 ng/mL, with the regression equation given by y = 62400x + 21300 (where y represents the peak area in the chromatogram and x is the pitolisant concentration). The correlation coefficient (*R* ^2^) was 0.999, and the limit of detection was 5 ng/mL.

Regression equation and detection limit of pitolisant in the brain: A strong linear relationship was observed for pitolisant concentrations ranging from 5 ng/mL to 1,000 ng/mL, with the regression equation given by y = 86331x - 1002712 (where y represents the peak area in the chromatogram and x is the pitolisant concentration). The correlation coefficient (*R* ^2^) was 0.997, and the limit of detection was 5 ng/mL.

Regression equation and detection limit of dopamine: A strong linear relationship was observed for dopamine concentrations ranging from 5 ng/mL to 5,000 ng/mL, with the regression equation given by y = 8,944x + 445,130 (where y represents the peak area in the chromatogram and x is the dopamine concentration). The correlation coefficient (*R* ^2^) was 0.999, and the limit of detection was 5 ng/mL.

## 4 Discussion

Pitolisant, a next-generation wakefulness-promoting agent, exerts its effects by antagonizing histamine H_3_ receptors, thereby promoting alertness and improving cognitive function. It has become a first-line treatment for narcolepsy. Compared to traditional psychiatric drugs, pitolisant has a more defined and singular mechanism of action, is non-addictive, and has fewer adverse effects, making it particularly suitable for specific activities such as shift work, rotating schedules, and certain military operations. However, its effectiveness in combating fatigue in these specific populations remains poorly supported by evidence. Additionally, HH environments can significantly affect the body’s metabolic processes and the functions of various organs, especially in regulating key physiological processes in the liver and nervous system. These changes may directly influence the ADME (absorption, distribution, metabolism, and excretion) processes of drugs, thereby altering their pharmacodynamic properties (Zhao et al., 2023). Despite its established efficacy under normoxic conditions, the pharmacodynamic effects of pitolisant in HH environments, particularly in high-altitude or military settings, have not been sufficiently studied.

In this study, we simulated HH environments using a small animal hypobaric chamber and administered varying doses (5 mg/kg, 20 mg/kg, and 40 mg/kg) of pitolisant via gavage. We assessed its impact on learning, memory, cognitive function, and motor coordination in sleep-deprived mice. The effects were systematically evaluated under both normoxic and HH conditions to compare fatigue resistance and pharmacodynamic differences. Furthermore, we used LC-MS/MS to analyze changes in pitolisant concentrations in mouse brain and plasma, measured dopamine levels in brain tissue from hypoxic mice, and investigated the expression of drug transporters P-gp and OCT1 to explore the potential mechanisms by which HH affects pitolisant’s pharmacodynamics.

Behavioral experiments (morris water maze, novel object recognition, and rotarod tests) revealed that pitolisant exhibited a clear dose-dependent effect under normoxic conditions. The 20 mg/kg dose showed significant pharmacodynamic effects across all behavioral tests. However, under the HH conditions, 20 mg/kg pitolisant failed to significantly improve the learning, memory, cognitive function, or motor coordination of the mice, while the 40 mg/kg dose still demonstrated good anti-fatigue effects. This suggests that HH significantly alters pitolisant’s pharmacodynamics, potentially due to changes in its ADME processes.

The results of drug concentration analysis in the brain and plasma support this hypothesis. Concentration-time curve analysis showed that HH significantly reduced pitolisant concentrations in the brain, especially during the absorption and distribution phases (0.25 h and 0.5 h post-administration). The rate of increase in brain concentration was slower, and the time to reach maximum brain concentration was delayed from 0.5 h (in the control group) to 1 h (in the HH group), suggesting that HH may inhibit pitolisant’s penetration of the blood-brain barrier (BBB). However, during the elimination phase (8 h post-administration), brain drug concentrations were significantly elevated. After 1 h of administration, blood concentrations in the HH group were significantly lower, and at the elimination phase (4 h and 16 h post-administration), blood concentrations were notably higher. These findings indicate that HH affects pitolisant’s absorption, distribution, metabolism, and excretion: HH significantly reduced the absorption and distribution processes while extending the metabolism and clearance phases. Under normoxic conditions, pitolisant exhibited the highest brain-to-plasma ratio of 3.875 at 4 h post-administration. During the peak concentration period (0.5 h) and at 4 h post-administration, the brain penetration of pitolisant in the HH group was significantly lower than that in the control group. These results explain the diminished pharmacodynamic effects of pitolisant under HH: the HH environment inhibited its ability to penetrate the BBB, thereby reducing its effective concentration in the brain and preventing the 20 mg/kg dose from achieving the expected pharmacological effect.

The concentration of drug in the brain is closely related to the expression of drug transporters at the BBB.P-gp, a key efflux transporter at the BBB, is a critical factor influencing the drug’s effective concentration in the brain (Chai et al., 2022; Shchulkin et al., 2024). OCT1, a transporter also present on BBB, plays a significant role in brain distribution of drugs (Doetsch et al., 2022; Romigi et al., 2018). We hypothesize that HH may alter the expression of P-gp and OCT1, thereby influencing the distribution of pitolisant in the brain. Our experimental data show that acute exposure to HH (1 h and 1 day) significantly reduced the protein expression of OCT1, which may lead to reduced brain penetration of pitolisant. Additionally, a 1-day exposure to HH significantly decreased both P-gp gene and protein expression, potentially delaying the efflux of pitolisant across the BBB. This mechanistic insight helps explain the biological basis of the pharmacodynamic changes observed under HH conditions. However, there is currently no evidence indicating that pitolisant is a substrate of any specific transporter; we can only speculate that P-gp and OCT1 may be involved in its absorption and transport processes. Future studies should further investigate and validate this hypothesis.

As an H_3_ receptor antagonist, pitolisant enhances cognitive function and alertness by modulating neurotransmitter release, thereby mitigating cognitive impairment caused by HH and sleep deprivation. The regulatory effect of histamine H_3_ receptors on dopamine, particularly in the brain’s nucleus accumbens, is a critical factor in evaluating drug addiction potential. To explore the impact of pitolisant on dopamine under HH conditions, we used HPLC-MS/MS to analyze dopamine levels in the brain of mice after 1 h, 1 day, and 3 days of pitolisant administration. The results revealed no significant changes in dopamine concentrations, confirming the safety of pitolisant in HH environments.

Although pitolisant retains its anti-fatigue effects under HH, its absorption, distribution, and pharmacodynamics have clearly altered. Therefore, these changes must be carefully considered when applying pitolisant in populations exposed to HH. Our study primarily discusses the effects of acute short-term HH exposure on pitolisant’s pharmacodynamics and its potential mechanisms. The duration of HH exposure may also have varying effects on pitolisant’s pharmacodynamics. Future research should investigate the pharmacodynamic characteristics and mechanisms of pitolisant under prolonged exposure to HH. Moreover, further studies are needed to assess the specific effects of HH on pitolisant’s metabolic pathways and explore the role of other transporters and drug-metabolizing enzymes in these pharmacodynamic changes.

## 5 Conclusion

In conclusion, we have, for the first time, clarified the anti-fatigue effect of pitolisant in sleep-deprived mice. Additionally, we have demonstrated that pitolisant improves learning and memory, cognitive function, and motor coordination in sleep-deprived mice exposed to HH. These findings provide experimental evidence for its potential application under HH conditions. Our results broaden the clinical application potential of pitolisant and highlight its promise in high-altitude regions where enhanced cognitive function is needed. However, further clinical data are required, particularly validation studies in populations with long-term HH exposure.

## Data Availability

The original contributions presented in the study are included in the article/Supplementary Material, further inquiries can be directed to the corresponding authors.

## References

[B1] Abdurakhmanova S. Grotell M. Kauhanen J. Linden A. M. Korpi E. R. Panula P. (2020). Increased sensitivity of mice lacking extrasynaptic δ-containing GABA(A) receptors to histamine receptor 3 antagonists. Front. Pharmacol. 11, 594. 10.3389/fphar.2020.00594 32435195 PMC7218123

[B2] Bonkowsky J. L. Son J. H. (2018). Hypoxia and connectivity in the developing vertebrate nervous system. Dis. Model Mech. 11 (12), dmm037127. 10.1242/dmm.037127 30541748 PMC6307895

[B3] Chai A. B. Callaghan R. Gelissen I. C. (2022). Regulation of P-glycoprotein in the brain. Int. J. Mol. Sci. 23 (23), 14667. 10.3390/ijms232314667 36498995 PMC9740459

[B4] Dauvilliers Y. Bassetti C. Lammers G. J. Arnulf I. Mayer G. Rodenbeck A. (2013). Pitolisant versus placebo or modafinil in patients with narcolepsy: a double-blind, randomised trial. Lancet Neurol. 12 (11), 1068–1075. 10.1016/S1474-4422(13)70225-4 24107292

[B5] Dauvilliers Y. Verbraecken J. Partinen M. Hedner J. Saaresranta T. Georgiev O. (2020). Pitolisant for daytime sleepiness in patients with obstructive sleep apnea who refuse continuous positive airway pressure treatment. A randomized trial. Am. J. Respir. Crit. Care Med. 201 (9), 1135–1145. 10.1164/rccm.201907-1284OC 31917607 PMC7193861

[B6] Doetsch D. A. Ansari S. Jensen O. Gebauer L. Dücker C. Brockmöller J. (2022). Substrates of the human brain proton-organic cation antiporter and comparison with organic cation transporter 1 activities. Int. J. Mol. Sci. 23 (15), 8430. 10.3390/ijms23158430 35955563 PMC9369162

[B7] Duan Y. Zhu J. Yang J. Gu W. Bai X. Liu G. (2021). A decade's review of miRNA: a center of transcriptional regulation of drugmetabolizing enzymes and transporters under hypoxia. Curr. Drug Metab. 22 (9), 709–725. 10.2174/1389200222666210514011313 33992050

[B8] Jala A. Ponneganti S. Vishnubhatla D. S. Bhuvanam G. Mekala P. R. Varghese B. (2021). Transporter-mediated drug-drug interactions: advancement in models, analytical tools, and regulatory perspective. Drug Metab. Rev. 53 (3), 285–320. 10.1080/03602532.2021.1928687 33980079

[B9] Kitanaka J. Kitanaka N. Hall F. S. Amatsu Y. Hashimoto K. Hisatomi E. (2020). *In vivo* evaluation of effects of histamine H(3) receptor antagonists on methamphetamine-induced hyperlocomotion in mice. Brain Res. 1740, 146873. 10.1016/j.brainres.2020.146873 32387137

[B10] Kotańska M. Mika K. Sałaciak K. Wheeler L. Sapa J. Kieć-Kononowicz K. (2020). Pitolisant protects mice chronically treated with corticosterone from some behavioral but not metabolic changes in corticosterone-induced depression model. Pharmacol. Biochem. Behav. 196, 172974. 10.1016/j.pbb.2020.172974 32565240

[B11] Krief S. Berrebi-Bertrand I. Nagmar I. Giret M. Belliard S. Perrin D. (2021). Pitolisant, a wake-promoting agent devoid of psychostimulant properties: preclinical comparison with amphetamine, modafinil, and solriamfetol. Pharmacol. Res. Perspect. 9 (5), e00855. 10.1002/prp2.855 34423920 PMC8381683

[B12] Langen U. H. Ayloo S. Gu C. (2019). Development and cell biology of the blood-brain barrier. Annu. Rev. Cell Dev. Biol. 35, 591–613. 10.1146/annurev-cellbio-100617-062608 31299172 PMC8934576

[B13] Meskill G. J. Davis C. W. Zarycranski D. Doliba M. Schwartz J. C. Dayno J. M. (2022). Clinical impact of pitolisant on excessive daytime sleepiness and cataplexy in adults with narcolepsy: an analysis of randomized placebo-controlled trials. CNS Drugs 36 (1), 61–69. 10.1007/s40263-021-00886-x 34935103 PMC8732895

[B14] Romigi A. Vitrani G. Lo Giudice T. Centonze D. Franco V. (2018). Profile of pitolisant in the management of narcolepsy: design, development, and place in therapy. Drug Des. Devel Ther. 12, 2665–2675. 10.2147/DDDT.S101145 PMC6124464 30214155

[B15] Shchulkin A. V. Abalenikhina Y. V. Kosmachevskaya O. V. Topunov A. F. Yakusheva E. N. (2024). Regulation of P-glycoprotein during oxidative stress. Antioxidants (Basel) 13 (2), 215. 10.3390/antiox13020215 38397813 PMC10885963

[B16] Uguen M. Perrin D. Belliard S. Ligneau X. Beardsley P. M. Lecomte J. M. (2013). Preclinical evaluation of the abuse potential of Pitolisant, a histamine H_3_ receptor inverse agonist/antagonist compared with Modafinil. Br. J. Pharmacol. 169 (3), 632–644. 10.1111/bph.12149 23472741 PMC3682710

[B17] Zhao A. Li W. Wang R. (2023). The influences and mechanisms of high-altitude hypoxia exposure on drug metabolism. Curr. Drug Metab. 24 (3), 152–161. 10.2174/1389200224666221228115526 36579391

[B18] Zolotoff C. Voirin A. C. Puech C. Roche F. Perek N. (2020). Intermittent hypoxia and its impact on Nrf2/HIF-1α expression and ABC transporters: an *in vitro* human blood-brain barrier model study. Cell Physiol. Biochem. 54 (6), 1231–1248. 10.33594/000000311 33326735

