# Supplementary material for: Acute hypobaric hypoxia attenuates the anti-fatigue effects of pitolisant by downregulating the expression of organic cation transporter 1 and P-glycoprotein
Source: Front Pharmacol. 2025 Apr 16;16:1564174. doi: 10.3389/fphar.2025.1564174 (PMC12041214; doi:10.3389/fphar.2025.1564174)
Supplement: Supplementary file 1 [file DataSheet1.docx]

1.Accuracy, Precision, and Recovery of pitolisant in plasma.

The within-day and between-day precision of pitolisant were both satisfactory, with values less than 9.61%. The accuracy varied between 1.62% and 8.34%. The recovery rate ranged from 96.83% to 108.34%. Stability testing results indicated that the samples remained stable under the specified conditions, with no significant degradation observed.

Supplementary Table 1. Evaluation of within-day accuracy, precision, and recovery（$\bar{X}$±s,n=6）

| Actual concentration(ng/mL) | Measured concentration(ng/mL) | Accuracy(％) | Precision (％) | Recovery(％) |
| --- | --- | --- | --- | --- |
| 10 | 10.178±0.306 | 3.00 | 1.78 | 101.78 |
| 100 | 107.382±4.694 | 4.37 | 7.38 | 107.38 |
| 1000 | 970.445±14.034 | 1.45 | 2.96 | 97.04 |

Supplementary Table 2. Evaluation of between-day accuracy, precision, and recovery（$\bar{X}$±s,n=6）

| Actual concentration(ng/mL) | Measured concentration(ng/mL) | Accuracy(％) | Precision (％) | Recovery(％) |
| --- | --- | --- | --- | --- |
| 10 | 9.683±0.931 | 9.61 | 1.62 | 96.83 |
| 100 | 108.337±10.098 | 9.32 | 8.34 | 108.34 |
| 1000 | 1013.11±95.609 | 9.44 | 1.62 | 101.31 |

Supplementary Table 3. Stability Evaluation of PitolisantPitolisant（$\bar{X}$±s,n=6）

| Storage conditions | Actual concentration(ng/mL) | Measured concentration(ng/mL) | Accuracy(％) | Precision (％) |
| --- | --- | --- | --- | --- |
| Room temperature（4 h） | 10 | 9.376±0.595 | 6.35 | 6.24 |
|  | 100 | 104.526±1.484 | 1.42 | 4.53 |
|  | 1000 | 983.867±8.256 | 0.84 | 1.61 |
|  | 10 | 9.522±0.854 | 8.96 | 4.78 |
| 4℃（24h） | 100 | 96.317±2.178 | 2.26 | 3.68 |
|  | 1000 | 1012.236±15.302 | 1.51 | 1.22 |
|  | 10 | 9.815±0.108 | 1.10 | 1.85 |
| -20℃（10days） | 100 | 92.574±1.223 | 1.32 | 7.43 |
|  | 1000 | 969.813±8.459 | 0.87 | 3.02 |
|  | 10 | 10.362±1.252 | 12.08 | 3.62 |
| -80℃（30days） | 100 | 105.923±5.430 | 5.13 | 5.92 |
|  | 1000 | 1088.333±96.351 | 8.85 | 8.83 |

2.Accuracy, Precision, and Recovery of pitolisant in brain.

The within-day and between-day precision of pitolisant were both satisfactory, with values less than 8.08%. The accuracy fluctuated between 1.24% and 7.37%. The recovery rate ranged from 92.63% to 106.53%. Stability testing results showed that the samples remained stable under the specified conditions, with no significant degradation observed.

Supplementary Table 4. Evaluation of within-day accuracy, precision, and recovery（$\bar{X}$±s,n=6）

| Actual concentration(ng/g) | Measured concentration(ng/g) | Accuracy(％) | Precision (％) | Recovery(％) |
| --- | --- | --- | --- | --- |
| 10 | 9.263±0.176 | 1.90 | 7.37 | 92.63 |
| 100 | 95.992±1.289 | 1.34 | 4.01 | 95.99 |
| 1000 | 1012.433±5.887 | 0.58 | 1.24 | 101.24 |

Supplementary Table 5. Evaluation of between-day accuracy, precision, and recovery（$\bar{X}$±s,n=6）

| Actual concentration(ng/g) | Measured concentration(ng/g) | Accuracy(％) | Precision (％) | Recovery(％) |
| --- | --- | --- | --- | --- |
| 10 | 10.624±0.382 | 3.59 | 6.24 | 106.24 |
| 100 | 97.315 ±7.861 | 8.08 | 2.68 | 97.31 |
| 1000 | 1065.318±62.521 | 5.87 | 6.53 | 106.53 |

Supplementary Table 6. Stability Evaluation of PitolisantPitolisant（$\bar{X}$±s,n=6）

| Storage conditions | Actual concentration(ng/g) | Measured concentration(ng/g) | Accuracy(％) | Precision (％) |
| --- | --- | --- | --- | --- |
| Room temperature（4 h） | 50 | 52.022±0.593 | 1.14 | 4.04 |
|  | 100 | 96.424±1.560 | 1.62 | 3.58 |
|  | 500 | 485.382±3.224 | 0.66 | 2.92 |
|  | 50 | 55.752±3.326 | 5.97 | 1.15 |
| 4℃（24h） | 100 | 97.922±7.910 | 8.08 | 2.08 |
|  | 500 | 475.232±20.183 | 4.25 | 4.95 |
|  | 50 | 46.316±1.908 | 4.12 | 7.37 |
| -20℃（10days） | 100 | 108.258±3.129 | 2.89 | 8.26 |
|  | 500 | 481.926±7.849 | 1.63 | 3.61 |
|  | 50 | 47.429±0.683 | 1.44 | 5.14 |
| -80℃（30days） | 100 | 96.726±0.418 | 0.43 | 3.27 |
|  | 500 | 478.472±3.657 | 0.76 | 4.31 |
